# High dose of nimustine as an add-on treatment for small cell lung cancer with intracranial metastasis: A case report and literature review: Retraction

**DOI:** 10.1097/MD.0000000000009328

**Published:** 2017-12-15

In the artice, “High Dose of Nimustine as an Adding on Treatment for Small-Cell Lung Cancer with Intracranial Metastasis: a Case Report and Literature Review”,^[1]^ which appeared in Volume 96, Issue 41 of *Medicine*, the authors of the paper have asked that their case report be retracted. During submission, the authors stated that they had received appropriate patient consent, however, the author group has notified the journal that they have failed to receive the patient's approval.
